# Bowel Movement: Integrating Host Mobility and Microbial Transmission Across Host Taxa

**DOI:** 10.3389/fmicb.2022.826364

**Published:** 2022-02-15

**Authors:** Arne Weinhold

**Affiliations:** Faculty of Biology, Cellular and Organismic Networks, Ludwig-Maximilians-Universität München, Munich, Germany

**Keywords:** gut microbiota, movement ecology, microbial dispersal, host movement, migration, environmental acquisition, community assembly, social microbiome

## Abstract

The gut microbiota of animals displays a high degree of plasticity with respect to environmental or dietary adaptations and is shaped by factors like social interactions, diet diversity or the local environment. But the contribution of these drivers varies across host taxa and our ability to explain microbiome variability within wild populations remains limited. Terrestrial animals have divergent mobility ranges and can either crawl, walk or fly, from a couple of centimeters toward thousands of kilometers. Animal movement has been little regarded in host microbiota frameworks, though it can directly influence major drivers of the host microbiota: (1) Aggregation movement can enhance social transmissions, (2) foraging movement can extend range of diet diversity, and (3) dispersal movement determines the local environment of a host. Here, I would like to outline how movement behaviors of different host taxa matter for microbial acquisition across mammals, birds as well as insects. Host movement can have contrasting effects and either reduce or enlarge spatial scale. Increased dispersal movement could dissolve local effects of sampling location, while aggregation could enhance inter-host transmissions and uniformity among social groups. Host movement can also extend the boundaries of microbial dispersal limitations and connect habitat patches across plant-pollinator networks, while the microbiota of wild populations could converge toward a uniform pattern when mobility is interrupted in captivity or laboratory settings. Hence, the implementation of host movement would be a valuable addition to the metacommunity concept, to comprehend microbial dispersal within and across trophic levels.

## Introduction

Microbial associations with animal hosts are ubiquitous and increasingly recognized as important factor for the understanding of host ecology and evolution (Shapira, 2016; Foster et al., 2017; Kolodny et al., 2020; Moeller and Sanders, 2020). The gut microbiota of host animals provides various important roles regarding digestion, provision or nutrients or immune stimulating function within insects as well as vertebrate clades (Moran et al., 2019; Schmidt and Engel, 2021). There is a huge interest in advancing our understanding on how host-associations are formed and how much inter-host transmission, environmental acquisition or host genetic factors play a role (Robinson et al., 2019; Mallott and Amato, 2021). While major focus is clearly on functional aspects for the host, reliable transmission routes and acquisition fidelity would sustain microbial associations even in the absence of mutualistic interactions (Leftwich et al., 2020; Sieber et al., 2021). For successful colonization and establishment as resident gut member, microbes must first encounter the host, which is increasingly more likely when one or both of the partners is mobile (Obeng et al., 2021). While microbial motility can be essential for the establishment of symbioses in aquatic and marine environments (Raina et al., 2019), terrestrial animals could directly alter the probability of encountering microbes by relocating themself. Host mobility range and frequency of movements differ across animal taxa, from highly mobile to more resident species.

Various studies applied a metacommunity concept for an eco-evolutionary understanding of host microbiome associations, including environmental microbes as a regional species pool (Adair and Douglas, 2017; Carrier and Reitzel, 2017; Miller et al., 2018). Host movement can extend these theoretical applications from a static toward a more dynamic acquisition process (Mihaljevic, 2012). Host aggregations reduces spatial scale and directly influence probability of inter-host transmissions, while host foraging movements could extend microbial acquisition range or increase microbial dispersal across different habitats.

While insects have (beside vertically transmitted obligate endosymbionts) relatively simple and species poor gut microbiotas (Colman et al., 2012; Engel and Moran, 2013; Jones et al., 2013), most mammals are known for a highly diverse and host taxon specific gut microbiota, that often fits to the phylogenetic distance of the host (the so called “phylosymbiosis” pattern) (Groussin et al., 2017; Groussin et al., 2020; Kohl, 2020). Though this is believed to be the result of an adaptive evolution of mammalian clades for dietary specialization, such a framework does not fit well to the microbiota of birds or bats, which show high inter individual dissimilarity and little influence of host phylogeny (Davenport et al., 2017; Lutz et al., 2019; Song et al., 2020).

With this manuscript I would like to highlight the role of host “movement” and mobility range as a neglected parameter explaining patterns in the microbiota of mammals, birds and insects and the potential transfer across host taxa.

## How Host Movement Increases Microbial Dispersal

**FIGURE 1:**
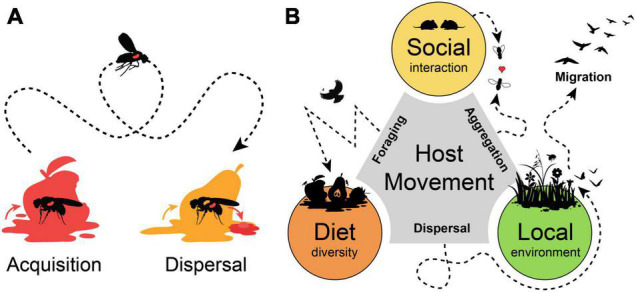
Host movement promotes microbial acquisition and dispersal. **(A)** Host movement influences microbial acquisition range of a host and promotes microbial dispersal across larger distances. **(B)** Host movement and mobility range influence major drivers of host microbiota. Host aggregation movement promotes social interactions and microbial transmission among hosts. Foraging movement range determines diet diversity of a host. Dispersal movement, nomadism or migration relocate a host into another location from where environmental microbes can be acquired.

By moving from one into another place animal hosts can vector microbes over a broad distance and increase their geographic distribution and dispersal rates. At the same time, a host can acquire different microbes from the new location, so that both effects of microbial *acquisition* and *dispersal* are often combined and hard to disentangle (Figure 1A). The vinegar fly *Drosophila melanogaster* is highly attracted by microbial volatiles and use this to find suitable oviposition sites within decaying and rotting fruits (Becher et al., 2012; Markow, 2015; Qiao et al., 2019). The highly mobile adults can disperse and vector microbes that accelerate the decaying process and support the development of the low-mobile larvae, which is a substantial aspect in the ecology of drosophilid and tephritid fruit flies (Wertheim et al., 2005; Wong et al., 2015; Pais et al., 2018). Under axenic conditions, adult flies respond with a restless behavior and increased locomotion activity (Schretter et al., 2018).

In pollination ecology, the high mobility and movement range of flying insects is a key factor to understand the dispersal abilities of the floral microbiota (Vannette and Fukami, 2017; Morris et al., 2019; Vannette, 2020). Pollinator foraging include repeated visitations of flowers by several different insects species, so that flowers can serve as hubs for microbial exchange within plant pollinator networks (Francis et al., 2021; Keller et al., 2021; Zemenick et al., 2021). Microbes can even directly influence pollinator behavior and preferences, altering floral visitation and nectar removal rates (Schaeffer et al., 2017; Rering et al., 2020; Jacquemyn et al., 2021). Similar, the distribution of multiple pollinator species drives parasite prevalence dynamics over the course of a season (Graystock et al., 2020). Like the common drone fly (*Eristalis tenax*), which promotes the dispersal of a hymenopteran gut parasite by contaminations of flower tissue with copious defecations (Figueroa et al., 2019; Davis et al., 2021).

## How Host Movement Shapes the Host Microbiota

A mobile host is not only a spreader, but also a receiver of microbes. Host animals can occupy various environmental niches, so that they can be associated with a diverse set of microbes (Carrier and Reitzel, 2017). The ecology and behavior of the host is an important aspect for host-microbial associations and their movement range would influence probabilities for microbial acquisition from different habitats or during social interaction (Ezenwa et al., 2012; Archie and Tung, 2015; Miller et al., 2018). Animals perform different kinds of movements, like non-directional “station-keeping movements” when foraging for food in a restricted area, or “dispersal movement” and “nomadism” as an erratic translocation across different habitats, until “migration” as a highly directional long-distance form of movement (Schlägel et al., 2020). All these movement behaviors could be drivers of the host microbiota, as they allow “social” interactions of a host with conspecifics, influence foraging range and “diet” diversity and determine if a host stays within a single “local” environment or frequently moves across diverse habitats (Figure 1B). Predictions for the outcome could be divergent and case specific, as host mobility could enhance similarities among social group structures within geographic isolated populations. While for other hosts, a high dispersal rate and foraging range could blur local influences and result in high dissimilarities among individuals from a single habitat. Thus, the application of a unified framework for all animal hosts would be challenging, as the influence of stochastic and deterministic factors change with the ecological context of the host and its mobility range (Sieber et al., 2019; Mallott and Amato, 2021).

### How Host Movement Defines the Microbial Acquisition Range

**FIGURE 2:**
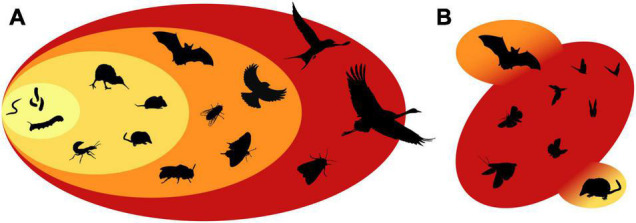
Host movement and mobility ranges across different host taxa. **(A)** Animals have diverse mobility ranges which could influence the probability for microbial acquisition and dispersal. Small animals or insect larvae (pale yellow) have limited movement abilities, while walking animals (yellow) can move into different environments and interact with other hosts. Flying animals (orange) can extend their foraging range to larger areas, while migrating species (red) can perform long-distance movements. **(B)** Potential extension of the microbial acquisition range across trophic levels. Insectivorous animals (i.e., bats or shrews) could acquire microbes from their prey (i.e., nocturnal moth) so that their microbiota would resemble more that of flying or migrating species.

Animals with limited movement abilities depend largely on the regional pool of microbes in their vicinity, which could enhance the correlation of host microbiota with sampling location (Figure 2A, pale yellow). This category includes mainly very small animals or insect larvae. A prominent member would be probably *Caenorhabditis elegans*, which is literally dwelling in microbial rich habitats as it can be found in nature on decomposing plant material and rotting fruits (Frézal and Félix, 2015). *C. elegans* is mainly relocated by vectoring animals and its gut microbiota composition is largely determined by the local substrate conditions, which can be distinct from that under laboratory conditions (Zhang et al., 2017). Even larger insect larvae (i.e., caterpillars) are mainly influenced by the foliar microbiota of their local environment, when remaining on a single host plant. Caterpillars are not known to depend on microbial associations nor enrichment of host-specific microbiota and interindividual variability can be explained by different host plants or collection sites (Hammer et al., 2019; Jones et al., 2019; Mason et al., 2020).

The second category contains animals that can perform terrestrial locomotion or “walking” (Figure 2A, yellow). This includes most mammals (including humans), flightless birds or ground-dwelling insects. Such movement abilities increase the mobility range and enable selective foraging for host-specific diets or allows social interactions, which would both increase microbiota similarity among conspecifics. In the wood mouse (*Apodemus sylvaticus*), the tracking of individual movement patterns revealed, that social connectivity and encounters with conspecifics explains microbiota similarity better than spatial distances or genetic relatedness (Raulo et al., 2021). In humans and primates, social group structures or “co-housing” within a shared living environment have a stronger influence on microbiome similarity among individuals than host-genetic factors or kinship (Tung et al., 2015; Rothschild et al., 2018; Brito et al., 2019; Robinson et al., 2019). Over larger geographic distances dispersal limitations and prey preferences explain gut microbiota dissimilarities among carnivorous mammals in allopatric populations (Moeller et al., 2017). While at a smaller geographic distance (< 25 km), the microbiota of wild mammals does not cluster by location, but mainly by host species identity and dietary preference (Knowles et al., 2019). Movement allows a selective foraging for specific diets within the same habitat, so that herbivorous rodents (mice and voles) are dominated by Bacteroidetes and Firmicutes, while their sympatric insectivorous neighbors (shrews) show increased abundances of Proteobacteria (Knowles et al., 2019).

The third movement category includes all “flying” animals (Figure 2A, orange), which contains most birds, bats as well as several flying insects. Active flight enlarges the range for microbial acquisition further and might dissolve signatures of local habitat boundaries. Characteristic for birds and bats is the low correlation of their microbiota with host phylogeny and a minor influences of sampling locality or habitat type (Hird et al., 2015; Lutz et al., 2019; San Juan et al., 2020; Bodawatta et al., 2021). Studies that have combined individual flight behavior of birds with host microbiota analysis are extremely rare, but for the barn owl (*Tyto alba*) the medium foraging range indicated a positive correlation with microbial diversity (Corl et al., 2020). Also insects showed typically high dissimilarities among individuals, low biogeographic patterns and only a weak influence of host phylogeny (Colman et al., 2012; Jones et al., 2013; Yun et al., 2014; Bahrndorff et al., 2017; Wang et al., 2020). But flying insects do not necessarily cover larger absolute geographic distances than terrestrial animals, but mobility *per se* with a high frequency of movements within a smaller range could expose a host to heterogeneous microenvironments. This is particularly important for microbe–plant–pollinator interactions, since the dispersal of nectar microbes is directly linked to the foraging behavior of pollinators (Cullen et al., 2021; Francis et al., 2021). Wild bee species vary in their foraging ranges from a few hundred meters to several kilometers, while nocturnal moth can easily exceed movement ranges to several hundred kilometers (Greenleaf et al., 2007; Satterfield et al., 2020).

The fourth movement category (Figure 2A, red) includes species that “migrate,” which differs from the previous movement categories, as it is a seasonal directional movement over larger geographic distance. Migration has been primarily investigated in birds and butterflies regarding the long-distance dispersal of parasites or as a strategy to avoid pathogen infections (Altizer et al., 2011; Bartel et al., 2011; Boulinier et al., 2016; Viana et al., 2016). But a few studies have investigated if host migration influences gut microbial diversity. While migrating passerine birds showed a change of their microbiota following a dietary shift (Lewis et al., 2017; Skeen et al., 2021), other studies pointed mainly at physiological adaptations and little environmental acquisition of microbes (Risely et al., 2017, 2018; Wu et al., 2018; Turjeman et al., 2020). The influence of migration on the microbiota of birds seems rather marginal and could be mainly attributed to dietary shifts between geographic distant locations. With insects, the influence of migration is less clear. Though, insect migration is often associated with the spectacular mass migration events of the monarch butterfly (*Danaus plexippus*) or the painted lady (*Vanessa cardui*), migration abilities are not uncommon for other butterflies or nocturnal moth, but remain often unnoticed as they fly in lower numbers or at night (Chowdhury et al., 2021). But even seasonal mass migration events are easily overlooked, when insects are small and fly at high altitudes like the marmalade hoverfly (*Episyrphus balteatus*) (Hu et al., 2016; Wotton et al., 2019; Satterfield et al., 2020). But to what extent insect migration would influence microbiota acquisition or dispersal has not been fully elucidated yet.

### Does ‘Flight’ or the Consumption of Mobile Insect Prey Shape the Host Microbiota?

A recent comparative study concludes that a convergent physiological adaption to “flight” (reduced dependence on microbes to reduce weight of the digestive system) shapes the bird as well as the bat microbiota (Song et al., 2020). Particular Proteobacteria seem somehow associated with “flight,” as they are commonly found in birds, bats and insects, while most mammals are dominated by Bacteroidetes and Firmicutes (Brooks et al., 2016; Song et al., 2020). But there are notable exceptions. In the analysis of Song et al. (2020) the order of Insectivora (here mainly shrews of the genus *Crocidura* spp.) showed the second highest proportions of Proteobacteria and a less mammal-specific microbiota than Chiroptera (bats), directly followed by Pholidota (ant-eating Pangolins). If the observed pattern would be explained by a physiological adaptation to flight, this raises the question why terrestrial shrews have the most “bird-like” microbiota of all mammals.

What is intriguing about this observation is the possibility that insectivorous mammals might obtain their microbiota directly from their prey. In such a scenario, the consumption of insects would expand their microbial acquisition range, which resembles more that of a flying insect (Figure 2B). A transfer of microbes across predator-prey networks has been suggested for insectivorous birds and predatory insects (Tiede et al., 2017; Suenami et al., 2019; Dion-Phénix et al., 2021). The mammalian microbiota is strongly influenced by species identity and type of diet, but an increase of invertebrate prey (i.e., insects) within the diet correlates with a decrease in bacterial alpha diversity compared to mammals with a primarily herbivorous lifestyle (Knowles et al., 2019; Harrison et al., 2021). Tough “insectivory” includes the consumption of non-flying insects (i.e., ants or termites) as well as other invertebrates, such a dietary preference seems to result in a convergent adaptation in the microbiota of phylogenetically distant mammalian clades (Pilosa, Cingulata, Tubulidentata, and Carnivora) (Delsuc et al., 2014). Particular bats might be able to further expand their microbial acquisition range beyond their own flight range, as they consume a diverse variety of highly mobile insect species within the orders Lepidoptera, Diptera, and Coleoptera (Tiede et al., 2020). The Brazilian free-tailed bat (*Tadarida brasiliensis*) preys on high altitude flying insects, which includes several migrating species, and noctuid moth make on average 77% of their diet (Krauel et al., 2018). There is clearly more work needed to clarify to what extend the microbiota of insects influences the microbiota of insectivorous animals across tropic levels (Figure 2B), and what patterns would be predicted for the microbiota of highly mobile hosts.

### Interruption of Host Movement Behavior in Captivity or Laboratory Settings

Captivity and laboratory settings can alter the outcome of microbiome studies tremendously and should be taken with caution when implying evolutionary context (Hird, 2017). As long as the insectivorous bat *Mops condylurus* preys on flying insects, they show higher interindividual variability with relative low alpha diversity in their fecal microbiota, but converge toward a more uniform community composition with increased alpha diversity when kept in captivity for 6 weeks (Edenborough et al., 2020). When brought into captivity, primates tend to shift toward a more “human-like” microbiota (Nishida and Ochman, 2021), which was attributed to a reduced diversity of food plants with lower fiber content compared to the naturally foraged diet of wild relatives (Clayton et al., 2016). Captivity has a significant effect on the microbiota of several mammal species (Kohl et al., 2014; McKenzie et al., 2017), and there is a lot potential to further explore how restrictions in foraging movement and range size shape the microbiome. Captivity alters also the microbiota of migratory as well as terrestrial birds, such as the crane and the brown kiwi (Xie et al., 2016; San Juan et al., 2021). Any restriction of animal movement behavior could disrupt natural host-microbial dispersal routes leading to more “uniform” results that confound the outcome obtained in laboratory settings or under captivity conditions.

## Conclusion and Future Perspectives

A major challenge in the investigation of the microbiota of wild animals is the lack of a clear framework of what can be expected when host species identity or geographic location are failing short to predict the observed variability within wild populations. By an integration of flight behavior, the study by Song et al. (2020) marks a transition that elevates from a pure host phylogenetic perspective toward the integration of host ecology. There is an exciting potential for future research to combine hypothesis about animal behavior and movement decisions with the host microbiota (Davidson et al., 2020; Bo and Kohl, 2021).

**FIGURE 3:**
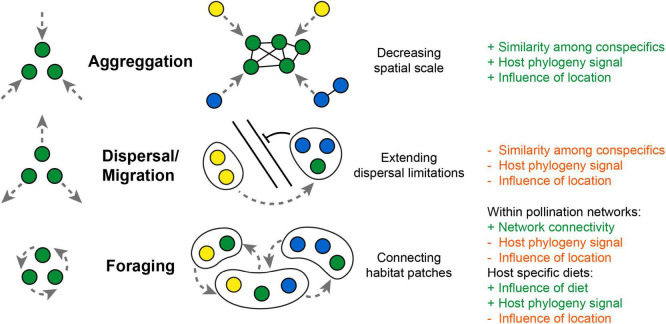
Potential framework how moving hosts (as “floating islands”) influence parameter of microbiota composition. Aggregation movement decreases spatial scale and promotes social exchange among conspecifics, which could increase correlation by host phylogeny. Dispersal movement could extend dispersal limitations of microbes but weakens the influence of local landscape parameters or host phylogeny. Foraging movement could connect habitat patches by distributing microbes across plant-pollinator networks and weakens influences of host phylogeny or the local environment. While a selective foraging for host specific diets (e.g., mammalian feeding guilds) would increase correlations with host diet and phylogeny (+ and – refers to an increase or decrease in correlation).

In the past, the analysis of host microbiomes has been mainly performed from a “host-centric” viewpoint, following the tradition of genetic model systems in the search of a host-genetic basis that explains microbiome composition. The inclusion of the “microbes perspective” has challenged this view and brings community ecological principles and stochastic processes into host microbiota analysis (Obeng et al., 2021; Sieber et al., 2021). The host microbiota is not a constant trait, but shows context dependent plasticity and interindividual variability in time and space (Carrier and Reitzel, 2017). Especially the metacommunity concept has become very useful in describing host-associated microbiota as it provides a framework that integrates interhost transmission and environmental acquisition of microbes from an external pool (Miller et al., 2018). As a reference to island biogeography, hosts are often depicted as passive “microbial habitats” that become colonized by microbes, similar as islands become colonized by other macrobiota, while social connectivity is illustrated by clustering single host “islands” into “archipelagoes” as used in the analogy by Sarkar et al. (2020). Though this mainly refers to stable social group structures, it misses to depict the dynamic and transient nature of these interactions. Moving hosts would be more comparable to “floating islands” which constantly change their spatial distribution relative to each other, actively connect in social interactions or relocate themselves into different environments (Figure 3).

Similar as microbial movement (motility) turned out to be an important aspect of host microbiota associations within aquatic systems (Raina et al., 2019), host movement (mobility) could become a vital addition to the metacommunity concept explaining the acquisition and dispersal of microbes among terrestrial animals. Movement changes several parameters that influence microbiome composition and could strength similarities among conspecifics via selective foraging or social exchange (Figure 3). Social transmission would become merely a consequence of aggregation movement, which decreases spatial scale and enhances the probability of microbial transmission among conspecifics. But movement could also blur correlations with host phylogeny and lead to higher dissimilarity among conspecifics from the same location. The mobility range and foraging pattern of a host could be a key factor to fully comprehend the composition and diversity of the host microbiota. Though a direct tracking of host movement in the wild is indeed a challenge, the outstanding work by Raulo et al. (2021) and Skeen et al. (2021) demonstrates the value of repeated sampling and the integration of movement patterns as a new dimension into microbiota analysis of birds and mammals. But even for insects, where a direct tracking of individuals is less feasible, the investigation of transmission routes within plant pollinator networks becomes a promising step to better understand microbial dispersal among different host taxa and across the animal and plant kingdom (Keller et al., 2021; Zemenick et al., 2021). Still, a framework for predictions of microbiota composition of highly mobile flying hosts is missing, as increasing complexity of interactions makes it difficult to directly correlate host mobility range with gut microbial diversity. Here, more work is needed to further explore if the same drivers can explain microbiota composition of flying hosts, resolving the patterns observed from insects, birds and bats.

## Data Availability Statement

The original contributions presented in the study are included in the article/supplementary material, further inquiries can be directed to the corresponding author/s.

## Author Contributions

AW conceived, designed, and wrote the manuscript.

## Conflict of Interest

The author declares that the research was conducted in the absence of any commercial or financial relationships that could be construed as a potential conflict of interest.

## Publisher’s Note

All claims expressed in this article are solely those of the authors and do not necessarily represent those of their affiliated organizations, or those of the publisher, the editors and the reviewers. Any product that may be evaluated in this article, or claim that may be made by its manufacturer, is not guaranteed or endorsed by the publisher.
